# Report of a Case of Rabies1Read before the Eleventh Annual Meeting of the Illinois Veterinary Medical and Surgical Association, Decatur, Ill., January 10 and 11, 1900.

**Published:** 1900-02

**Authors:** S. H. Swain

**Affiliations:** Decatur, Ill.


### REPORTS OF CASES.

### REPORT OF A CASE OF RABIES.[1]

By S. H. Swain, V.S.,
DECATUR, ILL.

On November 24, 1898, at about 8 P.M., I was called to the livery barn occupied by Thomas Doake, of Decatur, Ill., and on my arrival was informed by Mr. Doake that a horse owned by Michael McGinty, of this city, was sick and probably had colic. I at once proceeded to the stall where the horse was kept, and found a gray gelding lying on the floor, resting on the chest, with front limbs extended as in the act of rising; the horse was steaming; respiration rapid, with dilated nostrils, as though he had been exerting himself vigorously.

I directed Mr. Doake to make the horse rise, in order that I might give him a more thorough examination; a halter was put on him and he was called on to get up, which he endeavored to do, but failed in his posterior extremities. This effort revealed partial paralysis of the lumbar region, and yet the horse had fairly good use of the posterior limbs and tail; I then made a rather incomplete examination, observed the respiration, took the temperature per rectum, etc.; found the temperature 98.5°, and was undecided as to a correct diagnosis, but stated that the symptoms indicated cerebral and nervous derangement, and gave an unfavorable prognosis. I then administered a physic and a dose of nerve sedative.

[1] Read before the Eleventh Annual Meeting of the Illinois Veterinary Medical and Surgical Association, Decatur, Ill., January 10 and 11, 1900.

I next began an interrogation of Mr. Doake for a history of the case prior to my arrival, and was informed that the horse had been apparently well up to about 2 P.M. of that day, when the owner had driven him out of town a short distance, and was gone about three hours, returning at about 5 P.M., and on ariving home had notified Doake that the horse seemed warm, and that he would like him taken in and cared for at once, which was done, nothing unusual being observed; when the horse was sufficiently cooled out water and food were offered, which he refused. The man in charge at once informed Doake that something was the matter with the horse. Mr. Doake proceeded to investigate, and on arriving at the stall found the horse standing quietly, with head to the feed-box; he was approached from the rear and spoken to, and at this he wheeled around suddenly and made a vicious plunge at Mr. Doake, who stepped to one side, the horse's head and neck being at this time seized with a spasm, so that the mouth was drawn down to the sternum, the horse going down with a thud on the floor, from which he never arose. In the recital of the history of this case Mr. Doake remarked that Mrs. McGinty, the wife of the owner of this horse, had been bitten by a rabid dog which had made his home at the McGinty place, and that Mrs. McGinty had died of rabies about twelve months after she had been bitten. This bit of history, coupled with the symptoms, though I had never seen a rabid horse before this one, left but little doubt as to the case being rabies.

The patient was watched for a time, when he was left for the night, with the injunction by Doake that I be sure to call again early next morning, which I did, and on my arrival found the patient occupying exactly the same position as when I left him the night before. The first thing to attract my attention on this visit was that the horse had eaten every particle of bedding within his reach, and that the bedding was shavings from the planing-mill. Water was now offered him, and he shrank back from it as with disgust. Among the more prominent symptoms was a very hoarse cough, simulating croup, frequently repeated, with an attempt to neigh, as though lost from a companion. This act was also accompanied with a peculiar hoarseness and accomplished with difficulty. The pupils of the eyes were dilated and the eyes bore a very searching expression, as though watching for some object to appear. The nervous derangement was well marked by a rapid motion of the ears and tetanic symptoms of the muscles of the eyes, the left ear and eye being most affected. I might state that these symptoms were much more pronounced on my second visit than at the first.

After a careful observation of the above symptoms, on my second visit a correct diagnosis was easy and positively pronounced, and the owner informed to that effect, when the following additional facts were elicited from him: The horse had been bitten on the lip in October, 1896, by a rabid dog, the same dog having bitten Mrs. McGinty at about the time that he bit the horse. Mrs. McGinty developed the disease, from which she died on October 6, 1897, about one year after being bitten.

The history of this case shows that this most terrible disease had been lurking in the system of this horse for a period of two years and one month, or for a period of about seven hundred and sixty days before it manifested itself.

During my absence in the afternoon of the second day of the attack the horse became very furious, and was destroyed lest he do some harm. One of the peculiarities of this case is that the horse went down immediately after the first symptoms were observed, and was never able to rise again.

